# Sex Differences in Inflammation-Related Biomarkers Detected with OCT in Patients with Diabetic Macular Edema

**DOI:** 10.1016/j.xops.2024.100580

**Published:** 2024-07-18

**Authors:** Xinyi Chen, Wendy Yang, Ashley Fong, Noor Chahal, Abu T. Taha, Jeremy D. Keenan, Jay M. Stewart

**Affiliations:** 1 Department of Ophthalmology, University of California, San Francisco, San Francisco, California; 2 Department of Ophthalmology, Zuckerberg San Francisco General Hospital and Trauma Center, San Francisco, California; 3 Francis I. Proctor Foundation for Research in Ophthalmology, University of California, San Francisco, San Francisco, California; 4 Department of Radiology and Biomedical Imaging, University of California, San Francisco, California

**Keywords:** Biomarker, Diabetic retinopathy, Inflammation, OCT, Sex difference

## Abstract

**Purpose:**

To investigate sex-based differences in inflammation-related biomarkers on spectral-domain OCT.

**Design:**

Cross-sectional study.

**Participants:**

Patients with diabetic macular edema (DME) between February 1, 2019, and March 31, 2023, without intravitreal anti-VEGF injection within the previous 6 months.

**Methods:**

We reviewed each patient’s medical record for age, biological sex, race and ethnicity, most recent glycated hemoglobin A1c (HbA1c) level, visual acuity (VA), and central macular thickness (CMT). OCT biomarkers that have been found in literature to be associated with inflammation, including disorganization of retinal inner layers (DRIL), retinal hyperreflective retinal foci (HRFs), hyperreflective choroidal foci (HCFs), subfoveal neuroretinal detachment (SND), and perturbation in retinal nerve fiber layer thickness, ganglion cell layer thickness, and inner nuclear layer (INL) thickness were evaluated by graders masked to the clinical characteristics of the patients. We performed multivariable regression analyses with the OCT biomarkers as the outcome variables and sex, age, HbA1c, and CMT as independent variables.

**Main Outcome Measures:**

OCT inflammation-related biomarkers, as listed above.

**Results:**

Female patients were, on average, 2 years older than male patients (*P* = 0.041). There were no significant differences in race and ethnicity, HbA1c, VA, or CMT between male and female patients. After controlling for age, HbA1c, and CMT, we found male sex to be associated with more HRF (incidence rate ratio [IRR] = 1.19; 95% confidence interval [CI] = 1.10–1.29), more HCF (odds ratio = 2.01; 95% CI = 1.12–3.64), and thicker INL (7 μm thicker in males; 95% CI = 2–12). Sex was not a significant predictor for either DRIL or SND in the multivariable regression models. Patients with higher HbA1c were more likely to have more HRF (IRR = 1.02 per 1 point increase; 95% CI = 1.00–1.04) after controlling for other factors.

**Conclusions:**

Male sex was correlated with more inflammation-related biomarkers on OCT including more HRF, more HCF, and thicker INL, after accounting for age, glycemic control, and amount of DME. Further studies are needed to evaluate the potential implications of these sex-based differences for individualized treatment.

**Financial Disclosures:**

Proprietary or commercial disclosure may be found in the Footnotes and Disclosures at the end of this article.

Diabetic macular edema (DME) is a common vision-impairing complication of diabetic retinopathy (DR), affecting 5% to 10% of patients with diabetes mellitus (DM).^1^ Male sex has been found to be associated with a higher incidence of DR, a higher risk of progression of DR, and a higher chance of developing DME.^2^, ^3^, ^4^ The mechanisms underlying these sex-based differences remain unclear.

The pathogenesis of DR and DME is complex and involves manifold aberrant processes, but inflammation is thought to play a role in each condition.^5^ In DR, inflammatory cells such as resident microglia become activated, releasing cytokines and damaging neuronal and vascular endothelial cells. In DME, microglia are found to migrate into outer retina layers and subretinal space.^6^ Spectral-domain OCT (SD-OCT) is a high-resolution imaging modality of retinal anatomy. Several biomarkers on SD-OCT, such as disorganization of retinal inner layers (DRIL) and retinal hyperreflective retinal foci (HRFs), have been found in prior histologic studies to reflect activities of microglia and studies on both biofluid cytokines and OCT imaging features to be positively correlated with inflammation.^7^, ^8^, ^9^ These inflammation-related biomarkers on OCT may serve as surrogate markers of retinal inflammation, especially local retinal inflammatory changes.^10^ Variations in the OCT inflammation-related biomarkers may shed light on the clinically observed differences in DR between men and women.

The primary purpose of this study was to investigate sex differences in inflammation-related biomarkers of DME on SD-OCT. We hypothesized that male patients with DME would show higher levels of inflammation-related biomarkers on OCT compared to female patients.

## Methods

The study design was consistent with the tenets of the Declaration of Helsinki and was approved by the Institutional Review Board of the University of California, San Francisco, with waiver of the need for patient consent.

This retrospective study was conducted at the Zuckerberg San Francisco General Hospital and Trauma Center (ZSFG), an urban safety-net hospital affiliated with University of California, San Francisco and operated by the San Francisco Department of Public Health. To identify patients with DME deemed by providers to be significant enough to receive treatment, we searched the ZSFG electronic health record system for patients with the International Classification of Diseases, Ninth Revision codes and International Classification of Diseases 10 codes of DR (250.5∗, 362.0∗, E08.3∗, E09.3∗, E10.3∗, E11.3∗, E13.3∗) and a Current Procedural Terminology code of intravitreal injection (67 028) between February 1, 2019, and March 31, 2023. If multiple Current Procedural Terminology codes for intravitreal injection existed in the timeframe, the earliest encounter date was captured. The medical records of all identified patients were reviewed. Patients were included only if they were 18 years or older with a history of diabetes (type 1 or type 2) and had SD-OCT imaging (Spectralis; Heidelberg Engineering) on the date of or within a week prior to the date of their injection. Exclusion criteria included the following: (1) diagnosis with other vitreoretinal diseases in addition to DR or DME; (2) current or previous history of uveitis; (3) previous treatment including intravitreal anti-VEGF injection within the prior 6 months, intravitreal or peribulbar corticosteroids at any time prior, focal/grid macular photocoagulation at any time prior, panretinal photocoagulation at any time prior, or vitreoretinal surgery at any time prior; and (4) cataract surgery within the prior 6 months. To capture any additional care received before patients’ entry to the ZSFG system, we recorded any history of treatments that were documented in ZSFG providers’ notes from history taking. Patients with significant media opacity that interfered with OCT image acquisition (deemed by ≥2 of the graders to have insufficient quality for the assessment of the study features) were also excluded.

Each patient’s medical record was reviewed for age, sex, race and ethnicity, laterality of eye, glycated hemoglobin A1c (HbA1c) level (preferably within the prior 3 months; if not available within the prior 3 months, most recent value), visual acuity (VA) (pinhole or best corrected, when available), central macular volume (1-mm-radius zone around the fovea, calculated in Spectralis), and central macular thickness (CMT) (1-mm-radius zone around fovea, calculated in Spectralis). We included biological sex at birth in the study.

All images were acquired by trained ophthalmic photographers. A 20° × 15° (5.8 mm × 4.3 mm) area centered on the fovea was scanned with 19 B-scans and 9 automated real-time means per scan on the high-resolution mode. Enhanced depth imaging OCT was used to image the full thickness of the choroid. If both eyes received intravitreal injections, the OCT images of the eye with worse VA were included in the study.

### Image Analysis

**Figure 1:**
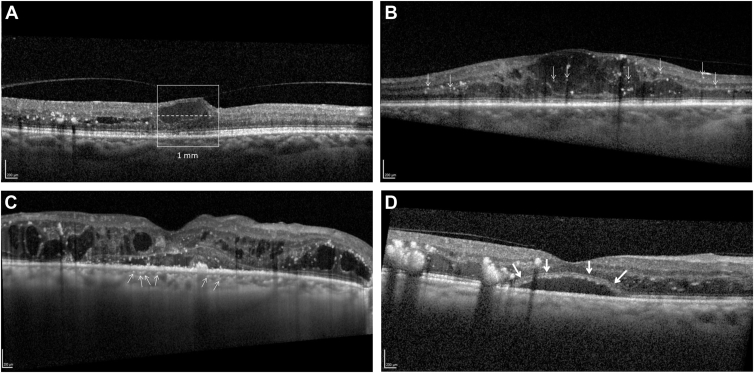
Representative examples of several inflammation-related biomarkers. **A,** Disorganization of retinal inner layers. The extent of disorganization of retinal inner layers (dashed line) was measured in the 1-mm-wide area centered on the foveal depression. **B,** Retinal hyperreflective retinal foci marked with white arrows. **C,** Hyperreflective choroidal foci marked with white arrows. **D,** Subfoveal neuroretinal detachment with margins marked by white arrows.

For each study eye, the scan passing through the foveal center was selected along with 1 scan immediately above and 1 scan immediately below, for a total of 3 scans (spaced 240 μm apart). OCT inflammation-related biomarkers analyzed in this paper included DRIL, HRF, hyperreflective choroidal foci (HCFs), subfoveal neuroretinal detachment (SND), retinal nerve fiber layer (RNFL) thickness, ganglion cell layer (GCL) thickness, and inner nuclear layer (INL) thickness. Examples of these biomarkers are provided in Figure 1.

Disorganization of retinal inner layers was defined as the horizontal extent in microns in a 1-mm-wide area centered on the foveal depression where the clear demarcation between the ganglion cell–inner plexiform layer complex, the INL, and the outer plexiform layer is lost (Fig 1A). The finding of DRIL is assessed independently of retinal edema. The DRIL extent was graded in each of the 3 B-scans, and these gradings were averaged across 3 scans to derive a global DRIL measure for each eye.

Hyperreflective retinal foci were defined as round, hyperreflective dots in the retina (Fig 1B). To distinguish them from hard exudates or microaneurysms, HRFs need to have similar reflectivity to RNFL and a diameter of less than 30 μm. The number of HRFs was counted in the entire scan and summed from the 3 B-scans for each eye.

Hyperreflective choroidal foci were defined as circumscribed dots within the choroid, having equal or higher reflectivity than the retinal pigment epithelium band (Fig 1C). The number of HCFs was counted in the enhanced depth imaging image.

Subfoveal neuroretinal detachment was defined as subfoveal hyporeflective area because of a neurosensory retinal detachment (Fig 1D). The area of SND was measured in mm^3^ in each of the 3 B-scans and averaged.

For patients with no or minimal DRIL, automated segmentation of retinal layers was performed in the Heidelberg software. If there was clear inaccuracy in the automated segmentation (inappropriate outline of optical reflectivity boundaries), manual segmentation was conducted instead.^11^ The mean thickness of RNFL, GCL, and INL layers in the pericentral area (1-mm diameter centered on the fovea) from the 3 scans in each eye was calculated.

A set of OCT images from a given eye was graded by 3 independent graders masked to the study identifier and clinical information, with the median value among the graders used in analyses. Operationally, the grading team consisted of 4 graders, of which 3 were randomly assigned to each set of OCT images. A separate set of 20 test images (i.e., images of the study population but from a different encounter date) were assessed by each masked grader 2 separate times to assess the repeatability of OCT grading. The intraclass correlation coefficient for intergrader reliability ranged from 0.66 to 0.99 for the inflammation-related OCT biomarkers. The intraclass correlation coefficient for intrarater reliability ranged from 0.70 to 0.92.

### Statistical Analysis

We performed statistical analyses using R, version 3.6.2. We compared patient characteristics and OCT biomarkers between men and women using chi-square tests for categorical variables and Wilcoxon rank sum tests for continuous variables. We used multivariable regression models with the inflammation-related biomarkers as the outcome variables and patient characteristics as independent variables. Because of the large number of zeroes for HCF and SND, they were categorized into present or absent for further analyses after being initially graded as continuous variables. The independent variables included in the model (sex, age, HbA1c, and CMT) were proposed in a priori hypotheses to be associated with OCT inflammation-related biomarkers. A generalized linear model was used for the count variable HRF. Logistic regression models were used to evaluate binary dependent variables such as HCF. Linear regression models were adopted for continuous dependent variables such as DRIL. To address the issue of multiplicity, we used a false discovery rate approach with the 2-stage step-up method of Benjamini, Krieger, and Yekutieli in the models.^12^ All statistical tests were two-tailed, with a *P* value of ≤0.05 as statistically significant.

## Results

**Figure 2:**
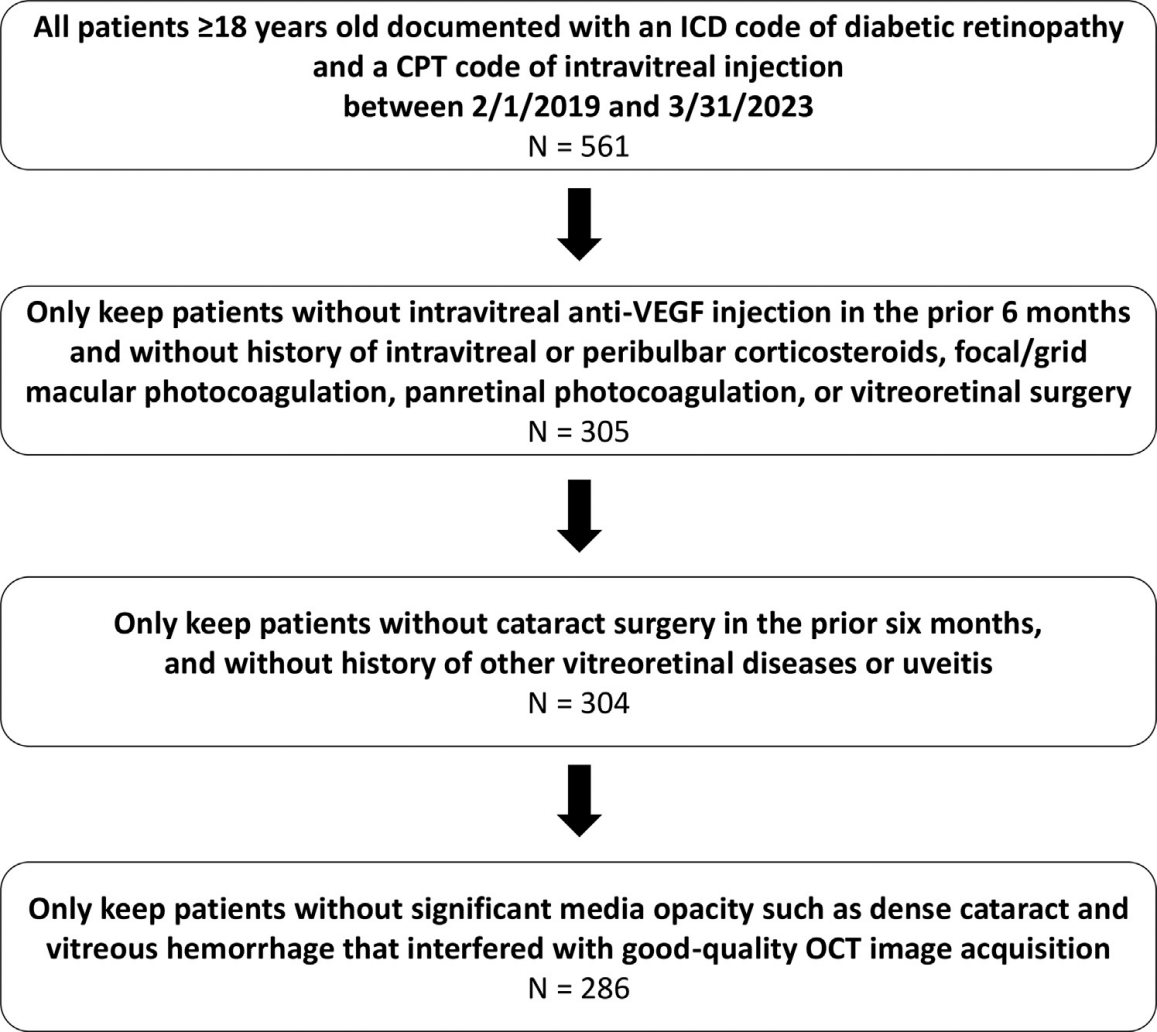
Flow diagram of patients included and excluded in the study. CPT = Current Procedural Terminology; ICD = International Classification of Diseases; OCT = Optical Coherence Tomography; VEGF = vascular endothelial growth factor.

A total of 561 patients met the inclusion criteria. After applying the exclusion criteria, we included 286 patients in the study, excluding 256 patients who had intravitreal anti-VEGF injection within the prior 6 months or a history of retinal laser or other vitreoretinal surgeries and excluding 18 patients because of poor-quality OCT images from significant media opacity (Fig 2). Among the included patients, 180 (62.9%) were male, and 106 (37.1%) were female. Female patients in this study were on average 2.1 years older than male patients (61.0 ± 10.5 years vs. 58.9 ± 10.6 years; *P* = 0.041). There was no significant difference in race and ethnicity between the male and female groups (Table 1). The mean HbA1c for all patients was 8.7% (standard deviation = 2.2%), with no significant difference between males and females. The average CMT of all included patients was 375.0 ± 135.1 μm. Male and female patients did not show a significant difference in VA or amount of DME (central macular volume or CMT).

**Table 1:** Demographic and Other Clinical Information

	Male (n = 180)	Female (n = 106)	*P* Value
Age (yrs), mean ± SD	58.9 ± 10.6	61.0 ± 10.5	0.041
Race and ethnicity, n (%)			
White∗	23 (12.8)	11 (10.4)	
Asian, Native Hawaiian, and other Pacific Islander∗	34 (18.9)	28 (26.4)	
Black or African American∗	20 (11.1)	16 (15.1)	
Hispanic	84 (46.7)	42 (39.6)	
Other	19 (10.6)	9 (8.5)	0.073
Laterality of eye, n (%)			
Right	98 (54.4)	50 (47.2)	
Left	82 (45.6)	56 (52.8)	0.286
Hemoglobin A1c (%), mean ± SD	8.5 ± 2.1	8.8 ± 2.3	0.232
Visual acuity†			
Median	20/40	20/50	
Range	20/20—HM	20/20—CF	0.332
Central macular volume (mm^3^), mean ± SD	9.76 ± 2.22	9.85 ± 1.89	0.259
Central macular thickness (μm), mean ± SD	375.6 ± 135.1	373.9 ± 135.9	0.601

CF = counting fingers; HM = hand motions; SD = standard deviation.

∗ Non-Hispanic.

† Pinhole or best-corrected visual acuity, if available, otherwise any visual acuity on the date of visit.

The OCT inflammation-related biomarkers for male and female patients are shown in Table 2. Female patients were found to have a larger extent of DRIL (493.5 ± 284.5 μm in male vs. 588.7 ± 279.6 μm in female; *P* = 0.006). Male patients had more HRF than female patients, although the result was not statistically significant in the bivariate analysis. We found that 96.3% of all eyes with HCF also had HRF. Compared with female patients, male patients had more HCF (1.2 ± 1.2 vs. 0.8 ± 1.1; *P* = 0.007), smaller SND (0.018 ± 0.095 vs. 0.020 ± 0.083 mm^2^; *P* = 0.039), and thicker INL in the pericentral area (44.6 ± 22.3 μm vs. 40.7 ± 26.4 μm; *P* < 0.001). No significant differences between male and female were found for the thickness of RNFL and GCL.

**Table 2:** Inflammation-Related Biomarkers on OCT

	Male	Female	*P* Values
DRIL (μm), mean ± SD	493.5 ± 284.5	588.7 ± 279.6	0.006
HRF, mean ± SD	13.7 ± 13.7	13.4 ± 12.2	0.785
HCF mean ± SD	1.2 ± 1.2	0.8 ± 1.1	0.007
0, n (%)	67 (37.2)	55 (51.9)	
>0, n (%)	113 (62.8)	51 (48.1)	0.022
SND (mm^2^) mean ± SD	0.018 ± 0.095	0.020 ± 0.083	0.039
0, n (%)	162 (90.0)	86 (81.1)	
>0, n (%)	18 (10.0)	20 (18.9)	0.051
RNFL thickness (μm), mean ± SD	21.5 ± 9.9	20.1 ± 7.0	0.229
GCL thickness (μm), mean ± SD	32.6 ± 11.0	31.8 ± 10.6	0.670
INL thickness (μm), mean ± SD	44.6 ± 22.3	40.7 ± 26.4	<0.001

DRIL = disorganization of retinal inner layers; GCL = ganglion cell layer; HCF = hyperreflective choroidal foci; HRF = hyperreflective retinal foci; INL = inner nuclear layer; RNFL = retinal nerve fiber layer; SD = standard deviation; SND = subfoveal neuroretinal detachment.

A summary of multivariable regression models is presented in Table 3. After controlling for age, HbA1c and CMT, male sex was associated with more HRF (incidence rate ratio = 1.19; 95% confidence interval [CI] = 1.10–1.29), more HCF (odds ratio [OR] = 2.01; 95% CI = 1.12–3.64), and thicker INL (7 μm thicker in males; 95% CI = 2–12). Sex was not a significant predictor for either DRIL or SND in the multivariable regression models. Patients with higher HbA1c were more likely to have more HRF (incidence rate ratio = 1.02 per 1 point increase in HbA1c; 95% CI = 1.00–1.04) after controlling for other factors.

**Table 3:** Multivariable Regression Models of Factors Associated with Inflammation-Related Biomarkers on OCT Images

	DRIL (μm)	
	Coefficient (95% CI)	*P* Value
Sex (male)	−68.37 (−142.75 to 6.00)	0.097
Age (decades)	−23.49 (−56.23 to 8.35)	0.148
HbA1c (%)	−15.65 (−32.05 to 0.74)	0.097
CMT (μm)	0.85 (0.58–1.12)	<0.001

	HRF (number)	
	Incidence Rate Ratio (95% CI)	*P* value
Sex (male)	1.19 (1.10–1.29)	<0.001
Age (decades)	0.92 (0.89–0.95)	<0.001
HbA1c (%)	1.02 (1.00–1.04)	0.031
CMT (μm)	1.00 (1.00–1.00)	<0.001

	HCF (presence)	
	Odds Ratio (95% CI)	*P* value
Sex (male)	2.01 (1.12–3.64)	0.039
Age (decades)	0.62 (0.47–0.82)	0.004
HbA1c (%)	1.00 (0.88–1.14)	0.989
CMT (μm)	1.00 (1.00–1.00)	0.989

	SND (presence)	
	Odds Ratio (95% CI)	*P* value
Sex (male)	0.38 (0.14–1.00)	0.104
Age (decades)	0.89 (0.59–1.37)	0.595
HbA1c (%)	1.08 (0.87–1.32)	0.595
CMT (μm)	1.01 (1.01–1.01)	<0.001

	RNFL thickness (μm)	
	Coefficient (95% CI)	*P* value
Sex (male)	0.94 (−2.02 to 3.89)	0.558
Age (decades)	−0.31 (−1.62 to 1.01)	0.558
HbA1c (%)	−0.54 (−1.19 to 0.11)	0.082
CMT (μm)	0.01 (0.00–0.02)	0.059

	GCL thickness (μm)	
	Coefficient (95% CI)	*P* value
Sex (male)	1.24 (−1.44 to 3.93)	0.487
Age (decades)	0.28 (−0.92 to 1.47)	0.653
HbA1c (%)	0.42 (−0.18 to 1.01)	0.338
CMT (μm)	0.03 (0.02–0.04)	<0.001

	INL thickness (μm)	
	Coefficient (95% CI)	*P* value
Sex (male)	6.90 (1.54–12.25)	0.025
Age (decades)	−0.02 (−2.41 to 2.36)	0.985
HbA1c (%)	0.21 (−0.98 to 1.39)	0.975
CMT (μm)	0.09 (0.07–0.11)	<0.001

CI = confidence interval; CMT = central macular thickness; DRIL = disorganization of retinal inner layers; GCL = ganglion cell layer; HbA1c = hemoglobin A1c; HCF = hyperreflective choroidal foci; HRF = hyperreflective retinal foci; INL = inner nuclear layer; RNFL = retinal nerve fiber layer; SND = subfoveal neuroretinal detachment.

## Discussion

Spectral-domain OCT is a noninvasive way to document inflammation-related changes in the retina. The findings in this study demonstrate that, in this population, male patients with DME had more inflammation-related biomarkers on OCT imaging than female patients. Male sex was correlated with more HRF, more HCF, and thicker INL in multivariable regression analyses controlling for age, HbA1c, and CMT.

In chronic inflammatory diseases such as DM, retinal glial cells become activated and release inflammatory mediators, causing neuronal cell and vascular endothelial cell damage.^6^ Hyperreflective retinal foci have been hypothesized to represent aggregates of activated microglia. Histologic studies on human eyes pointed to the origin of HRF from both anteriorly migrated retinal pigment epithelial cells and lipid-filled cells, which represent activated microglial cells.^10^ Alternatively, HRF have been hypothesized to represent lipid exudates or degenerated photoreceptors. However, previous studies have documented an increase in number of HRF in patients with early stages of DR or even diabetic patients without DR, challenging the explanation that HRF might represent lipid exudates or degenerated photoreceptors.^13^ In our study, we had specific criteria for HRF to distinguish them from lipid exudates and microaneurysms.

Hyperreflective retinal foci are associated with both local and systemic inflammation. Increases in cytokines expressed in microglia and macrophages such as CD14 were observed in aqueous humor in eyes with more HRF.^14^ Furthermore, increased HRF are associated with greater systemic inflammatory indices such as neutrophil-lymphocyte ratio and platelet-lymphocyte ratio.^7^ A reduction in HRF is observed after anti-VEGF treatment, and an even more significant reduction is seen after dexamethasone intravitreal implant, which inhibits chemotaxis and proliferation of microglial cells.^7^^,^^15^, ^16^, ^17^, ^18^ When patients had more baseline HRF, they experienced limited vision improvement after anti-VEGF treatment.^19^, ^20^, ^21^ In contrast, when patients with more HRF at baseline were treated with dexamethasone instead, more reduction in macular edema and greater improvement in retinal sensitivity on microperimetry were found.^16^^,^^17^^,^^22^ The result in the current study that compared to female patients with DME, male patients had more HRF suggests increased microglial accumulation and activity in males even with similar control of blood glucose and similar amount of DME. It is possible that because of increased HRF at presentation, male patients could be on average more responsive to corticosteroid treatment, although more studies are needed to confirm this potential therapeutic implication.

Our data also found male patients with DME to have more HCF when the glycemic control and the amount of DME were accounted for. Hyperreflective choroidal foci have been proposed as a continuum of HRF. The location of microglial cells varies depending on the severity of DR. Resident microglial cells are initially located in inner retinal layers and near ganglion cells. In fact, they are only found in the inner retina and are completely absent from the outer retina in healthy patients without DM. As the inflammatory process persists, microglial cells spread posteriorly, and more HRF are observed in outer retinal layers with more severe DR.^13^ The external limiting membrane (ELM) normally acts as a barrier preventing outward migration of inner retinal macromolecules. Roy et al showed that, with intact ELM/ellipsoid zone (EZ), eyes with DME could have HRF, but most did not have HCF.^23^ On the other hand, the disruption of ELM/EZ was observed in most eyes with HCF in addition to HRF. The loss of integrity of ELM/EZ permits the movement of microglia from retina to choroid and possibly the engulfment of degenerated photoreceptors by these microglia along the way.^24^ The majority of eyes in our study with HCF also had HRF, supporting the hypothesis that HCF and HRF are on a spectrum. The increased HCF in male patients implies increased microglial migration, likely because of inflammatory mediators and the breakdown of the ELM barrier. Similar to HRF, HCF on baseline OCT were correlated, with improved responsiveness to corticosteroid treatment in prior studies.^25^

Our study also found increased INL thickness in male patients with DME compared to female patients. Previous studies have documented an increase in INL thickness in patients with DR compared to controls.^26^ The main components of INL are nuclei of Müller cells and bipolar cells. Müller cells normally facilitate the communication between retinal vessels and neurons, maintain the metabolic homeostasis of the retinal extracellular environment, and establish the blood-retinal barrier.^27^ In response to hyperglycemia and hypoxia, Müller cells release inflammatory mediators and exhibit impaired fluid-absorbing capabilities.^27^ Under these pathological conditions, activated Müller cells are also observed to undergo hypertrophy and hyperplasia on histologic studies.^6^^,^^28^ This corresponds to increased thickness in INL on OCT imaging. The increase in INL in male patients with DME versus females when systemic glycemic control and CMT were controlled for further corroborates the greater extent of retinal glial cell modification and activation.

A recent study has found a more inflammatory intraocular fluid environment in male patients versus female patients,^29^ and inflammatory proteins contribute to retinal neuronal cell apoptosis in DM.^30^ In patients with DM and no DR, men had higher levels of 12 inflammatory proteins in aqueous humor than women, including 7 chemokines, 2 proteases, 2 proteins involved in programmed cell death, and a T-cell surface protein. These findings imply the presentation of a more inflammatory phenotype in male patients with DM than female patients.^29^ Although biomarkers in aqueous humor and vitreous humor are highly correlated, studies of aqueous humor offer less proximity to the retina. Our current study takes a closer look at the local changes in the retina, especially those reflecting inflammatory glial cell activation. These findings suggest that increased inflammation in men might contribute to the clinically observed sex differences in DR and DME, although more studies are needed to confirm this.

Several hypotheses have been proposed regarding the mechanism for sex-based differences in the onset and severity of DR. Sex hormones are thought to be implicated. Estrogen seems to reduce retinal vessel resistance and protect against the impairment of blood flow in ocular diseases. Although many women in our study were postmenopausal, it has been found that the accumulative exposure to estrogen over the lifetime from menarche to menopause offers protective effects for ocular vascular diseases.^31^ On the other hand, testosterone levels are associated with the development and progression of DR.^32^^,^^33^ Another explanation for the sex-based differences concerns behavioral factors such as increased rates of substance use and unhealthy diets with greater proinflammatory potential among men compared to women.^34^^,^^35^ Further studies are needed to investigate the association of behavioral factors with inflammation manifested on biomarkers on imaging.

One other notable finding in our study is the positive correlation between HbA1c values and HRF numbers. This is consistent with prior studies that showed more HRF in patients with DME with higher HbA1c at baseline.^7^ This increase in HRF lasted 1 year after anti-VEGF therapy in one previous study. Additionally, studies have found more HRF in patients with early stages of DR without DME when they have poorer glycemic control,^36^ suggesting that the effect of HbA1c on microglial aggregates is independent of DME, consistent with our finding of positive correlation of HbA1c with HRF even after controlling for the degree of DME.

This study was limited, first, by its retrospective nature. Therefore, we could not control all possible confounders. We did try to control for clinical features such as HbA1c and amount of DME (CMT), but the overall severity of DR could not be accurately assessed and controlled for. The clinic notes were templated and pulled data from clinicians’ updates under the Problem List in the medical record system, and the DR severity specified in the problem title or International Classification of Diseases coding might not have been updated at each visit. We also considered grading fundus photos for DR severity as part of the study, but patients do not get fundus photos taken at every clinic visit. Because of the retrospective nature of our study, most patients had fundus photography at a different time point (usually during teleretinal screening, at initial clinic presentation, or for other ocular comorbidities) instead of during the encounter with the OCT images included in the study. Their DR could have progressed or regressed in severity in the meantime. Clinical information such as DM duration was also not readily available in the electronic medical records. A prior study of ours on a similar diabetic patient population conducted at the ZSFG included DM duration either from chart review or from self-reporting as part of the technician screening process in that study protocol, and the female patients were found to have longer durations of diabetes than males in this population.^37^ It is therefore less likely that men in our study population had more inflammation-related biomarkers on OCT because of longer durations of DM. Another limitation of this study is the manual counting of HRF and HCF, which could be subject to error, although we established good reliability among the graders before the grading of study images, and we used the median count from the graders for each biomarker for each patient in the analyses. Third, in patients with bilateral DME, we included the eye with worse VA in the study, which could have introduced selection bias.

The strength of the current study lies in the systematic masked evaluation of multiple OCT-based inflammation-related biomarkers in male versus female patients. Our results showed more inflammation-related OCT biomarkers in men, which suggests a more inflammatory phenotype of DR in men and potentially a need for closer follow-ups. As HRF and HCF on baseline OCT were correlated with improved responsiveness to corticosteroid treatment in prior studies, there might be a role for a lower threshold for other treatment failures in men to switch to steroid injections for DME, especially when inflammation-related biomarkers are observed on OCT. Future studies are needed to evaluate the potential implications of these sex-based differences of inflammation-related biomarkers on clinical outcomes and on tailored treatment.
